# An app to keep: smartphone-based dispatch of community first responder to cardiac arrest

**DOI:** 10.1186/s12872-025-04586-y

**Published:** 2025-03-07

**Authors:** Tore Marks, Bibiana Metelmann, Peter Brinkrolf, Karl Christian Thies, Klaus Hahnenkamp, Camilla Metelmann

**Affiliations:** 1 Department of Anaesthesiology, University Medicine Greifswald, Ferdinand-Sauerbruch Straße, 17489 Greifswald, Germany; 2 Department of Anaesthesiology, EvKB, Universitätsklinikum OWL der Universitaet Bielefeld, Campus Bielefeld-Bethel, EvKB, Bielefeld, Germany

**Keywords:** Out-of-hospital cardiac arrest, First responder, Citizen responder, Smartphone, Technology acceptance

## Abstract

**Background:**

Smartphone-based alerting of community first responders to out-of-hospital cardiac arrest (OHCA) is associated with enhanced survival. Community first responders are volunteers, who are dispatched by the emergency dispatch centre, if they are in close proximity to an OHCA to decrease time until first chest compression. For a community first responder system to be successful, it is essential to recruit and retain as many qualified community first responders as possible. This study evaluates the appraisal and retention rate of an app-based community first responders system over a period of 3 years.

**Methods:**

A longitudinal study among community first responder in a rural northern Germany was conducted using an online-survey. A questionnaire (7 open questions, 22 single choice questions and 2 multiple choice questions) was distributed to all community first responders (FR) via e-mail in October 2018, 2019 and November 2020. Ethical approval was obtained, informed consent was given by all participants.

**Results:**

The response rate was 69%, 43% and 38% in the first, second and third year, respectively. Three years after implementing the system 96% of the users stated they still had the app installed. After the first year, 21% of participants observed improvements. In the second year, this number was 15%, and 31% in the third year. The opinion regarding the medical benefit of the app was stable. Nine out of ten participants would recommend the app to others. Of all participants 70% identified as male and 66% were 35 years old or younger. Main barrier to using the app was excessive „battery consumption“.

**Conclusions:**

The community first responder system attracts a predominantly young and male user base. The retention rate of 96% over the three years observation period is high. The main barrier to app usage is excessive battery consumption. The users’ positive perceptions regarding the app’s medical advantages and the favorable perception of its functionality have resulted in a steadfast high recommendation rate.

**Supplementary Information:**

The online version contains supplementary material available at 10.1186/s12872-025-04586-y.

## Background

In cardiac arrest, early resuscitation is vital [1, 2]. During out-of-hospital cardiac arrests (OHCA), there is an unavoidable time lag between alerting emergency systems and the arrival of the Emergency Medical Service (EMS) [3, 4]. Even with ambulance response within five minutes, survival is poor without immediate cardiopulmonary resuscitation (CPR) [4, 5, 6, 7]. Several global programmes have trained laypersons in CPR, but bystander response remains limited [8, 9, 10, 11, 12, 13, 14]. To address this delay and support bystanders, many regions introduced first responder systems [15, 16, 17, 18, 19, 20, 21]. When first responders are nearby during an OHCA, they are alerted alongside ambulances [20]. Such systems help expedite CPR [16, 22], leading to enhanced survival and neurological outcomes [17, 23, 24, 25]. Regions with these systems report improved cardiac arrest survival rates [26]. The European Resuscitation Council and the American Heart Association recommend to implement first responder systems [27, 28]. Internationally, different hardware and software solutions are used to alert first responder through a smartphone app. Additionally, there are two different approaches to recruit first responder [29]. One approach is to dispatch crews of firefighters or police officers during their shifts [30]. The other approach is to recruit volunteers, who act as community first responder independent of their working shifts [31]. Community first responder can decide, whether they attend the mission and rush to the OHCA patient. While some community first responder systems recruit volunteers regardless their medical knowledge, other systems require resuscitation training or even limit their system to community first responder with medical background [32].

For a community first responder system to be successful, it is essential to recruit and retain as many community first responders as possible [20]. However, in our fast-paced world, smartphone applications (apps) are often dismissed quickly [33, 34]. Most apps lose more than half of their users within the first week [34]. User acceptance is thought to depend on several aspects, but foremost on perceived experience and appropriateness to user’s context and needs [35]. As a high user acceptance is associated with high retention rates, it is crucial for a functional first responder system to find out how satisfied users are with the apps. The existing literature in this field is sparse. Longitudinal studies are still pending.

Thus the aim of this study is to evaluate the users’ perception of the app, barriers to utilization and retention rates over a period of 3 years.

## Methods

### Background

In September 2017 a smartphone-based alert of community first responders to attend OHCA was launched in Vorpommern-Greifswald, a rural area in northern Germany. An app called “Land|Retter” was made available free of charge in both major app stores (Google Play Store and Apple App Store). Participation as first responder was voluntary. To participate, users had to (i) register and provide information on their qualification regarding resuscitation skills and (ii) attend a 2-hour seminar. Qualification could include employment in health care (e.g. physician, nurse, paramedic, medical student) or regular basic life support training (e.g. as a firefighter). The seminar focused on practical, medical, technical and legal aspects of app usage and included hands-on CPR-training. During the seminar first responders had to consent (i) to participate in the system, and could consent (ii) to anonymous mission data analysis, and (iii) to being contacted and invited for surveys.

### Description of the app

The Emergency Dispatch Center can activate the app “Land|Retter” 24/7 in addition to the regular EMS response if an OHCA is suspected. The system automatically alerts registered community first responders, who are coincidentally in close vicinity of the OHCA patient. The radius of activation around an OHCA is 750 m in urban and up to 2000 m in rural areas. FR are not alerted in case of potential danger at the emergency site (e.g. fire, violence, traffic accident). First responders are alerted by sound and message and can choose, whether they accept or decline this mission. During the study period up to two first responders could accept per mission. After accepting the mission, FR have to insert a pin code to start the navigation to the target site. FR can choose to cancel the mission at any point. Once they arrive at the emergency site, a metronome is activated to support chest compressions with the correct compression rate. During the study period 20 updates were made to improve functionality of the app software.

### Description of the questionnaire

To assess the system`s appraisal and retention rate a questionnaire was devised. As no validated questionnaires could be identified for this matter, a new questionnaire was developed and evaluated in a pretest regarding comprehensibility and feasibility. The questionnaire contains 31 questions (7 open questions, 22 single choice questions and 2 multiple choice questions). The online survey tool “Surveymonkey” (Survey-Monkey Inc., San Mateo, CA, USA) was used. All active FR (of whom a declaration of consent was available at the time of the survey) were contacted via e-mail. Invitations to participate were sent in October 2018, 2019 and November 2020. A reminder email was sent in all three years to all non-replying study participants one, two and three weeks after the initial survey invitation.

As recruiting of first responder continued between October 2018 and November 2020 the number of FR increased over time. Study participation was voluntary and without monetary or other compensation.

### Statistics

The study was designed as a longitudinal survey study. As the aim was to conduct a complete survey, a power analysis was not necessary.

Descriptive statistics were used for all participants with mean, standard deviation, median, relative and absolute frequencies. Statistical analysis was carried out using SPSS Statistics version 25.0.0.0 for Mac OS (SPSS Inc., Chicago, IL, USA).

## Results

Invitations to participate in the survey were sent to all first responders (in total 816 invitations in three years). Table 1 shows the response rate, which dropped from 69% (*n* = 126) in year one to 43% (*n* = 124) in year two to 38% (*n* = 129) in year three.

**Table 1:** Number of invited FR, number of responses shown in absolute numbers (n) and response rate shown in percentage (%)

	Year 1	Year 2	Year 3
Number of invited FR	182	290	344
Number of responses	126	124	129
Response rate	69%	43%	38%

### Participants‘ characteristics

In the aggregated data of all three years, 70% (*n* = 244) of participants identified as male. 66.2% of the first responder were 35 years old or younger, 24.1% were between 36 and 50 years old and 9.7% were older than 51 years. When asked about their qualification 35.3% stated „ambulance service“, 20.3% answered „fire fighter“, 22% specified being a „medical student“, and 22.4% had other qualifications. 54% (*n* = 199) of the FR stated using an android phone.

Apart from their duties as FR, 28.7% had never performed CPR before, 15.8% had only performed it once, 12.3% had performed CPR two to five times, and 43.3% had performed it more than five times.

### Evaluation by first responders

**Fig. 1:**
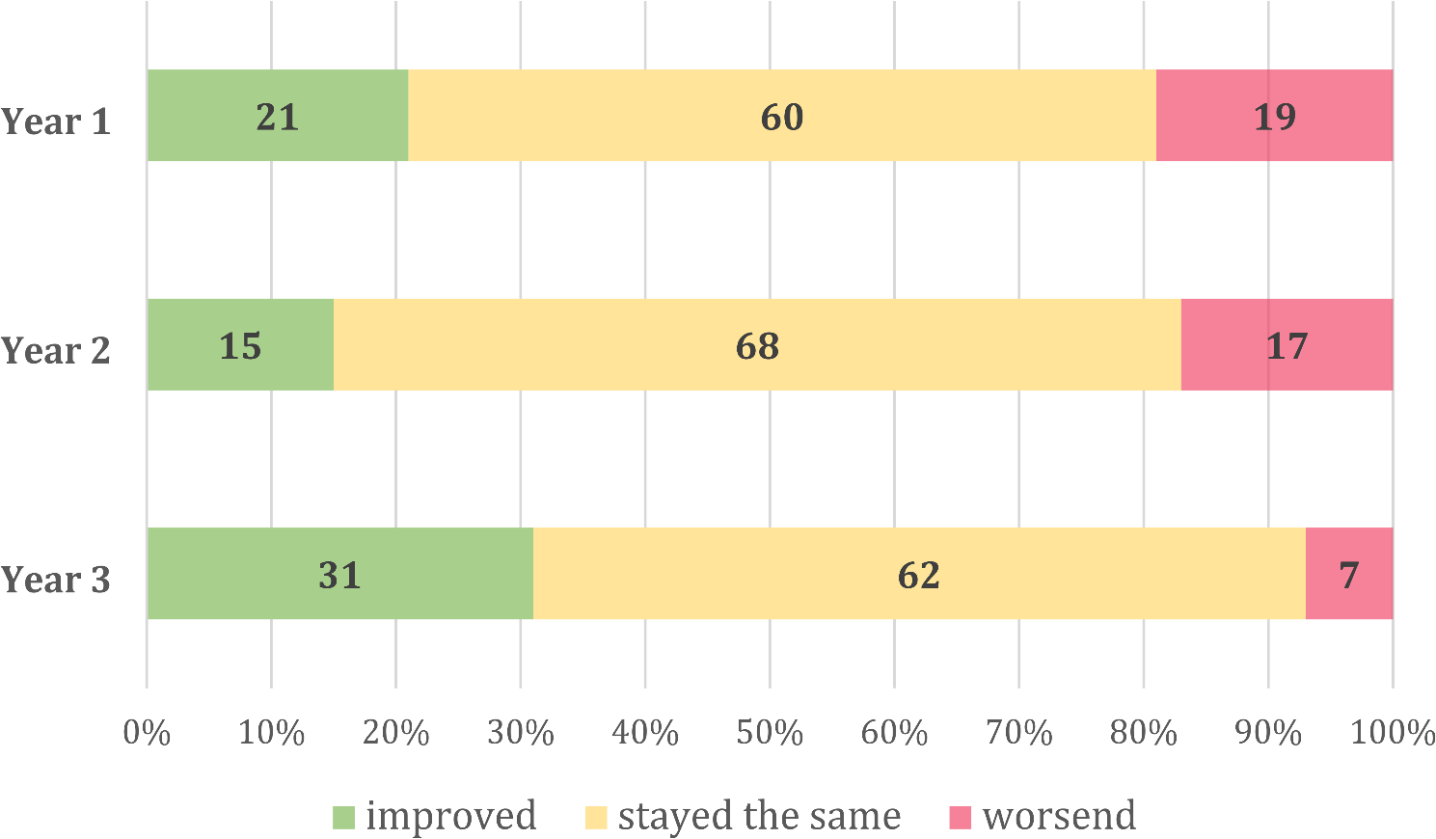
Attitude regarding the functionality of the app. Shown in percentage

**Fig. 2:**
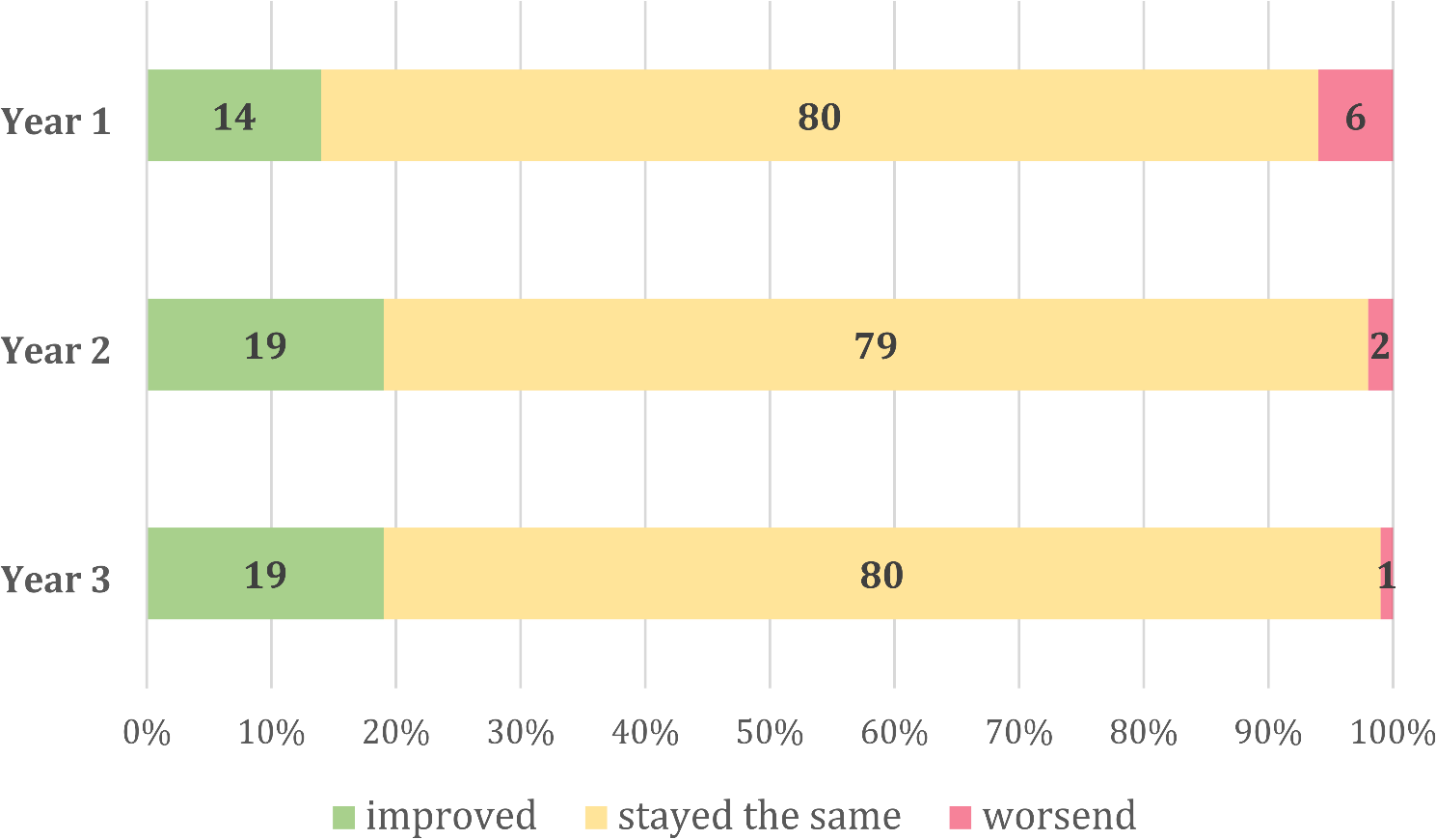
Attitude regarding the medical benefit of the app. Shown in percentage

Participants were asked how their opinion on the functionality and the medical benefit of the app changed over time, see Figs. 1 and 2.

First responders can decide whether to accept or decline a mission alert. In year one 43% (*n* = 23) indicated, that they had declined a mission. In year two 42% (*n* = 31) and in year three 63% (*n* = 57) reported to have declined a mission.

Table 2 presents the reasons for mission declinations.

**Table 2:** Answers to the question: “why did you decline a mission?” (multiple answers possible), shown in absolute numbers (n)

	Year 1	Year 2	Year 3
Malfunction of the app	5	7	9
The mission was not available any more	13	17	32
Deployment during working hours	9	10	21
Deployment in an inappropriate situation (e.g. going to the cinema/meeting)	4	10	18
I was looking after minors	4	2	10
I was unwell	2	3	8
I was concerned of not finding the mission site	0	2	0
I had drunk alcohol	0	5	6
I could not interrupt my activity	4	6	12
I was concerned of making a mistake during the mission	0	1	4
Other	1	3	12

### Retention and recommendation rate

One year after implementing the system 97% (*n* = 122) of the participants still had the app installed. After two years 98% (*n* = 121) and after three years 96% (*n* = 123) had retained the app. Nine out of ten participants recommended the app to others: 84% (*n* = 100) in year one and 91% (*n* = 109) and 90% (*n* = 107) in year two and three.

### Barriers to use the app

Table 3 shows barriers to using the app.

**Table 3:** Answers regarding the question: “are there barriers to using the app?” (multiple answers possible), shown in absolute numbers (n)

	Year 1	Year 2	Year 3
No reasons	61	76	68
Battery drainage	39	25	21
Data security	4	3	3
Mental stress caused by the permanent possibility to get dispatched	8	6	9
Concerned to get alarmed at an inconvenient time	20	28	32
Doubts regarding medical benefit	3	0	2
Legal aspects	3	0	2
Other	14	9	10

## Discussion

### Recommendation rate, functionality and medical benefit

By surveying the Land|Retter app users repeatedly, we were able to profile detailed user characteristics and also compare user behaviour over a period of three years. Throughout these three survey years, the proportion of first responders who retained the Land|Retter app remained stable at 97%, 98%, and 96% respectively. This is noteworthy considering the general tendency among smartphone users to employ most of their apps for only a short duration, with merely 4% continuing usage beyond 15 days [36, 37, 38].

The percentage of first responders recommending the app to others rose from 84% in the first year to 91% in the succeeding year, sustaining a robust recommendation rate of 90% in the third year. Despite already favourable initial values, the recommendation rate remained stable over the span of these three years. The perception of the first responders regarding the app’s functionality did not deteriorate over time. After Year 1, results showed that 21% of participants perceived improvements. In Year 2, 15% of participants reported improvements, followed by 31% in Year 3. During the first year after the launch iOS users could not be alerted whilst their phone was in silent mode. The update in the second year to override the silent mode has proved effective, as evidenced by a rising percentage of first responders who noted improved functionality. Other first responder systems experienced similar problems: The users of the Belgian “EVapp” interviewed by Vercammen et al. reported alert malfunctions in silent mode. The developers of the British app “GoodSAM” also recognized the problem and were able to implement suitable modifications in the alarm system [20, 39].

Despite technical challenges, the users’ attitude towards the medical benefit of the first responder app appears to remain consistently good over three survey years. The percentage of first responders stating that their opinion on the medical benefit of the app decreased dropped from 6% to 2% and 1% in the first, second and third year respectively.

Wade et al. demonstrated the crucial role of acceptance of a telemedicine application for a successful implementation. Through interviewing Australian users of telemedicine applications they found that staff acceptance is the foremost positive predictor for successful deployment of a telemedical application. If users are enthusiastic about the concept, they are less likely to be deterred by obstacles such as technical problems or funding issues [40].

### First responder characteristics

It is noteworthy that 7 out of 10 first responders who answered the questionnaire identified as male. International comparative studies implicate that more than two-thirds of first responders who register and perform CPR are male [41, 42]. A plausible explanation for the gender distribution among the first responders can be discerned when evaluating the qualifications. A significant 55.6% of first responder indicated “ambulance service” or “fire brigade“ as their qualification. The predominance of male gender in these professional groups could elucidate the accumulation of the male gender among the volunteers.

The majority (66.2%) of first responders who completed the questionnaire were aged below 36 years. The high proportion of young FR is in line with the fact that the prevalence of smartphone app usage tends to diminish in the older population [43]. Additionally, social commitment underlies “age-specific differences” [44]. Younger individuals tend to engage in volunteer emergency services and volunteer fire services more often than older individuals and are thus more likely to participate in first responder systems [45]. This trend is mirrored in the age distribution among users of first responder apps [46] and can be confirmed by the data collected about the Land|Retter App.

### Barriers to app utilisation

The survey consistently reveals over a three year period that battery drainage emerges as the foremost obstacle for first responders. Latency and energy depletion contribute to frustration significantly impacting app retention and abandonment rates [33].

Another major obstacle perceived by first responders is the concern of being alerted at an inconvenient time. However, only a few first responders reported declining a mission due to being alerted at such a moment. This suggests that their concerns about being alerted at inopportune times may not be fully justified. Although Phung et al. propose that the anxiety of being alerted at an inconvenient moment can be alleviated through regular training, this concern is challenging to eliminate entirely [47]. This highlights other advantages of recruiting qualified first responders: firefighters and EMS personnel are accustomed to the on-call nature of their roles. Plus, studies indicated that victims of OHCA would feel safer if resuscitated by a qualified first responder [48]. An implementation of fixed, schedulable absence times in the app could serve to prevent alerts during inconvenient moments.

Before participating in the described first responder system, individuals must complete comprehensive training covering technical, medical and legal aspects. Notably, over the course of three years, only a total of 5 first responders expressed concerns about legal aspects. This data emphasises the effectiveness of the provided training as a preparatory measure.

Over the span of three survey years, only five of the first responders cited a lack of medical meaningfulness as an obstacle to using the app.

### Reasons to decline a mission

The Land|Retter App operates on a voluntary base; hence declined calls are neither reported nor recorded. Information regarding these missions can only be gathered from the questionnaires. Over the course of three survey years, the percentage of first responders who stated that they had previously declined an alarm rose from 43 to 63%. The longer users participate, the higher is the number of alarms per user and the likelihood of having to decline a mission at some point increases.

Ozcan et al. explored the reasons why first responders might have to refuse to respond to an alarm, categorizing them into four barriers that need to be overcome: (1) “Barriers to commitment”, (2) „Barriers to notification“, (3) „Barriers to leave“, and (4) „Barriers to perform“ [49]. The methodological approach of the survey provides nuanced reasons for refusing an intervention. Most of which align with the four categories delineated in Ozcan’s group model. As survey participants were only individuals, who already committed to being a first responder, the first category “Barriers to commitment” was not assessed.

Unfortunately, alerts that failed to reach the first responder during an emergency due to, for instance, lack of network coverage, could not be accounted for in the first responder survey. Hence, the precise number of missions not accepted due to disruptions in the notification chain remains an estimation. Two barriers to notification were assessed: “mission was not available anymore” and “malfunction of the app”: In the first year 13 first responders reported not being able to accept an assignment because it was not available – a figure that rose to 17 in the second year and further to 32 in the third year. The system alerts all first responders in the vicinity of an OHCA simultaneously. The first two responders to accept the mission, receive the assignment, while others receive the notification that the mission is no longer available. An increasing density of first responders could explain the rising number of first responders for whom the operation was no longer available.

The third category “Barriers to leave” encompassed response options as “deployment during working hours”, “deployment in an inappropriate situation (e.g. going to the cinema/meeting)”, “I was looking after minors”, “I was unwell” and “I had drunk alcohol”. All reasons were cited more frequently in the third year than in the first year.

Over the span of four years, only five first responders reported refusing an assignment due to concerns of making a mistake. This underscores the confidence of the first responders in their own abilities and aligns with Ozcan’s assertion, that the fourth category (“Barriers to perform”) make up the smallest group among the reason for mission rejections.

Given the varying technical specifications and alarm modalities across different first responder systems, crafting a uniform, detailed questionnaire to ascertain reasons for mission refusal proves challenging. However, adopting a classification into groups, as proposed by Ozcan et al., appears to be a viable approach for future surveys.

### Limitations

The response rate exhibited a decline from 69% in the first year to 43% in the second year and further to 38% in the third year while noting an increased number of total first responders due to ongoing recruiting within the system. This poses challenges for comparative statistical analysis. Despite the decline, a response rate of 38% in the third year remains a commendable participation for online surveys, when compared internationally [50, 51]. The diminishing response rate could be attributed, for instance, by the repetitiveness of the surveys – using almost identical surveys consecutively over three years. First responders who participated in previous years may find their motivation waning. Moreover, there is no requirement to formally exit the Land|Retter system if one no longer wishes to receive alerts. Likewise, relocations out of the region is rarely reported back to the administrators. This also contributes to a scenario where, with the escalating total numbers of registered first responders, the proportion of first responders who can be alerted in the district of Vorpommern-Greifswald is lower than the total number of registered first responders, thereby distorting the response rate. Another limitation lies in the low external generalisability. Given the substantial disparities among smartphone based first responder systems, transferring data becomes a complex challenge.

Furthermore non-responder bias has to be considered [52]. Non-respondents are less likely to use the app or answer a questionnaire potentially skewing the results and overestimating its retention rate and functionality.

## Conclusions

Systems, that alert community first responders to OHCA by smartphone application, rely on a well-functioning app and need to retain as many first responders as possible over a long time span. By surveying community first responders using the Land|Retter app over a period of three years we identified excessive battery consumption and the apprehension of receiving alerts in an inconvenient moment as the main hindrances to its usage. Notwithstanding the technical hurdles, users’ perceptions regarding the app’s medical advantages have remained consistently positive.

This favorable perception of its functionality has resulted in a high retention and recommendation rates. In essence, users seem to view the Land|Retter app as precisely what it was designed to be: an app to keep.

## Electronic supplementary material

Below is the link to the electronic supplementary material.

Supplementary Material 1

## Data Availability

No datasets were generated or analysed during the current study.

## Abbreviations

APP: Application
CPR: Cardiopulmonary resuscitation
ERC: European Resuscitation Council
EMS: Emergency Medical Service
FR: First responder
GRC: German Resuscitation Council
OHCA: Out-of-hospital cardiac arrests

## References

[1] Soar J, Böttiger BW, Carli P, Couper K, Deakin CD, Djärv T, et al. European resuscitation Council guidelines 2021: adult advanced life support. Resuscitation. 2021;161:115–51. 10.1016/j.resuscitation.2021.02.010. 33773825 10.1016/j.resuscitation.2021.02.010

[2] Sasson C, Rogers MAM, Dahl J, Kellermann AL. Predictors of survival from out-of-hospital cardiac arrest: A systematic review and meta-analysis. Circ Cardiovasc Qual Outcomes. 2010;3:63–81. 10.1161/CIRCOUTCOMES.109.889576. 20123673 10.1161/CIRCOUTCOMES.109.889576

[3] Bürger A, Wnent * J, Bohn A, Jantzen T, Brenner S, Lefering R, et al. The effect of ambulance response time on survival following Out-of-Hospital cardiac arrest: an analysis from the German resuscitation registry. Dtsch Arztebl Int. 2018;115:541–8. 10.3238/arztebl.2018.0541. 30189973 10.3238/arztebl.2018.0541 PMC6156551

[4] Park GJ, Song KJ, Shin SD, Lee KW, Ahn KO, Lee EJ, et al. Timely bystander CPR improves outcomes despite longer EMS times. Am J Emerg Med. 2017;35:1049–55. 10.1016/j.ajem.2017.02.033. 28237384 10.1016/j.ajem.2017.02.033

[5] Hasselqvist-Ax I, Riva G, Herlitz J, Rosenqvist M, Hollenberg J, Nordberg P, et al. Early cardiopulmonary resuscitation in out-of-hospital cardiac arrest. N Engl J Med. 2015;372:2307–15. 10.1056/NEJMoa1405796. 26061835 10.1056/NEJMoa1405796

[6] Rajan S, Wissenberg M, Folke F, Hansen SM, Gerds TA, Kragholm K, et al. Association of bystander cardiopulmonary resuscitation and survival according to ambulance response times after Out-of-Hospital cardiac arrest. Circulation. 2016;134:2095–104. 10.1161/CIRCULATIONAHA.116.024400. 27881566 10.1161/CIRCULATIONAHA.116.024400

[7] Neukamm J, Gräsner J-T, Schewe J-C, Breil M, Bahr J, Heister U, et al. The impact of response time reliability on CPR incidence and resuscitation success: A benchmark study from the German resuscitation registry. Crit Care. 2011;15:R282. 10.1186/cc10566. 22112746 10.1186/cc10566 PMC3388696

[8] Deakin CD. The chain of survival: not all links are equal. Resuscitation. 2018;126:80–2. 10.1016/j.resuscitation.2018.02.012. 29471008 10.1016/j.resuscitation.2018.02.012

[9] Bohn A, Lukas RP, Breckwoldt J, Böttiger BW, van Aken H. Kids save lives’: why schoolchildren should train in cardiopulmonary resuscitation. Curr Opin Crit Care. 2015;21:220–5. 10.1097/MCC.0000000000000204. 25922895 10.1097/MCC.0000000000000204

[10] Mathiesen WT, Bjørshol CA, Kvaløy JT, Søreide E. Effects of modifiable prehospital factors on survival after out-of-hospital cardiac arrest in rural versus urban areas. Crit Care. 2018;22:99. 10.1186/s13054-018-2017-x. 29669574 10.1186/s13054-018-2017-x PMC5907488

[11] Ong MEH, Perkins GD, Cariou A. Out-of-hospital cardiac arrest: prehospital management. Lancet. 2018;391:980–8. 10.1016/S0140-6736(18)30316-7. 29536862 10.1016/S0140-6736(18)30316-7

[12] Riva G, Ringh M, Jonsson M, Svensson L, Herlitz J, Claesson A, et al. Survival in Out-of-Hospital cardiac arrest after standard cardiopulmonary resuscitation or chest compressions only before arrival of emergency medical services: nationwide study during three guideline periods. Circulation. 2019. 10.1161/CIRCULATIONAHA.118.038179. 30929457 10.1161/CIRCULATIONAHA.118.038179

[13] Shimamoto T, Iwami T, Kitamura T, Nishiyama C, Sakai T, Nishiuchi T, et al. Dispatcher instruction of chest compression-only CPR increases actual provision of bystander CPR. Resuscitation. 2015;96:9–15. 10.1016/j.resuscitation.2015.07.009. 26206594 10.1016/j.resuscitation.2015.07.009

[14] Gräsner J-T, Wnent J, Herlitz J, Perkins GD, Lefering R, Tjelmeland I, et al. Survival after out-of-hospital cardiac arrest in Europe - Results of the EuReCa TWO study. Resuscitation. 2020;148:218–26. 10.1016/j.resuscitation.2019.12.042. 32027980 10.1016/j.resuscitation.2019.12.042

[15] Scquizzato T, Burkart R, Greif R, Monsieurs KG, Ristagno G, Scapigliati A, Semeraro F. Mobile phone systems to alert citizens as first responders and to locate automated external defibrillators: a European survey. Resuscitation. 2020. 10.1016/j.resuscitation.2020.03.009. 32251700 10.1016/j.resuscitation.2020.03.009

[16] Berglund E, Claesson A, Nordberg P, Djärv T, Lundgren P, Folke F, et al. A smartphone application for dispatch of Lay responders to out-of-hospital cardiac arrests. Resuscitation. 2018;126:160–5. 10.1016/j.resuscitation.2018.01.039. 29408717 10.1016/j.resuscitation.2018.01.039

[17] Caputo ML, Muschietti S, Burkart R, Benvenuti C, Conte G, Regoli F, et al. Lay persons alerted by mobile application system initiate earlier cardio-pulmonary resuscitation: A comparison with SMS-based system notification. Resuscitation. 2017;114:73–8. 10.1016/j.resuscitation.2017.03.003. 28268186 10.1016/j.resuscitation.2017.03.003

[18] Hasselqvist-Ax I, Nordberg P, Herlitz J, Svensson L, Jonsson M, Lindqvist J, et al. Dispatch of firefighters and Police officers in Out-of-Hospital cardiac arrest: A nationwide prospective cohort trial using propensity score analysis. J Am Heart Assoc. 2017. 10.1161/JAHA.117.005873. 28978527 10.1161/JAHA.117.005873 PMC5721830

[19] Ringh M, Rosenqvist M, Hollenberg J, Jonsson M, Fredman D, Nordberg P, et al. Mobile-phone dispatch of laypersons for CPR in out-of-hospital cardiac arrest. N Engl J Med. 2015;372:2316–25. 10.1056/NEJMoa1406038. 26061836 10.1056/NEJMoa1406038

[20] Valeriano A, van Heer S, Champlain F, de C, Brooks S. Crowdsourcing to save lives: A scoping review of bystander alert technologies for out-of-hospital cardiac arrest. Resuscitation. 2021;158:94–121. 10.1016/j.resuscitation.2020.10.035. 33188832 10.1016/j.resuscitation.2020.10.035

[21] Oving I, Masterson S, Tjelmeland IBM, Jonsson M, Semeraro F, Ringh M, et al. First-response treatment after out-of-hospital cardiac arrest: a survey of current practices across 29 countries in Europe. Scand J Trauma Resusc Emerg Med. 2019;27:112. 10.1186/s13049-019-0689-0. 31842928 10.1186/s13049-019-0689-0 PMC6916130

[22] Sarkisian L, Mickley H, Schakow H, Gerke O, Jørgensen G, Larsen ML, Henriksen FL. Global positioning system alerted volunteer first responders arrive before emergency medical services in more than four out of five emergency calls. Resuscitation. 2020. 10.1016/j.resuscitation.2019.12.010. 31923531 10.1016/j.resuscitation.2019.12.010

[23] Scquizzato T, Belloni O, Semeraro F, Greif R, Metelmann C, Landoni G, Zangrillo A. Dispatching citizens as first responders to out-of-hospital cardiac arrests: a systematic review and meta-analysis. Eur J Emerg Med. 2022;29:163–72. 10.1097/MEJ.0000000000000915. 35283448 10.1097/MEJ.0000000000000915

[24] Pijls RWM, Nelemans PJ, Rahel BM, Gorgels APM. Characteristics of a novel citizen rescue system for out-of-hospital cardiac arrest in the Dutch Province of Limburg: relation to incidence and survival. Neth Heart J. 2019;27:100–7. 10.1007/s12471-018-1215-0. 30560444 10.1007/s12471-018-1215-0 PMC6352615

[25] Jonsson M, Berglund E, Baldi E, Caputo ML, Auricchio A, Blom MT, et al. Dispatch of volunteer responders to Out-of-Hospital cardiac arrests. J Am Coll Cardiol. 2023;82:200–10. 10.1016/j.jacc.2023.05.017. 37438006 10.1016/j.jacc.2023.05.017

[26] Oving I, de Graaf C, Masterson S, Koster RW, Zwinderman AH, Stieglis R, et al. European first responder systems and differences in return of spontaneous circulation and survival after out-of-hospital cardiac arrest: A study of registry cohorts. Lancet Reg Health - Europe. 2020;100004. 10.1016/j.lanepe.2020.100004. 10.1016/j.lanepe.2020.100004 PMC8454711 35104306

[27] Berg KM, Cheng A, Panchal AR, Topjian AA, Aziz K, Bhanji F, et al. Part 7: systems of care: 2020 American heart association guidelines for cardiopulmonary resuscitation and emergency cardiovascular care. Circulation. 2020;142:S580–604. 10.1161/CIR.0000000000000899. 33081524 10.1161/CIR.0000000000000899

[28] Semeraro F, Greif R, Böttiger BW, Burkart R, Cimpoesu D, Georgiou M, et al. European resuscitation Council guidelines 2021: systems saving lives. Resuscitation. 2021;161:80–97. 10.1016/j.resuscitation.2021.02.008. 33773834 10.1016/j.resuscitation.2021.02.008

[29] Grasner J-T, Bray JE, Nolan JP, Iwami T, Ong MEH, Finn J, et al. Cardiac arrest and cardiopulmonary resuscitation outcome reports: 2024 update of the Utstein Out-of-Hospital cardiac arrest registry template. Resuscitation. 2024;201:110288. 10.1016/j.resuscitation.2024.110288. 39045606 10.1016/j.resuscitation.2024.110288

[30] Nordberg P, Jonsson M, Forsberg S, Ringh M, Fredman D, Riva G, et al. The survival benefit of dual dispatch of EMS and fire-fighters in out-of-hospital cardiac arrest May differ depending on population density–a prospective cohort study. Resuscitation. 2015;90:143–9. 10.1016/j.resuscitation.2015.02.036. 25790753 10.1016/j.resuscitation.2015.02.036

[31] Gregers MCT, Andelius L, Kjoelbye JS, Juul Grabmayr A, Jakobsen LK, Bo Christensen N, et al. Association between number of volunteer responders and interventions before ambulance arrival for cardiac arrest. J Am Coll Cardiol. 2023;81:668–80. 10.1016/j.jacc.2022.11.047. 36792282 10.1016/j.jacc.2022.11.047

[32] Metelmann C, Metelmann B, Kohnen D, Brinkrolf P, Andelius L, Böttiger BW, et al. Smartphone-based dispatch of community first responders to out-of-hospital cardiac arrest - statements from an international consensus conference. Scand J Trauma Resusc Emerg Med. 2021;29(29). 10.1186/s13049-021-00841-1. 10.1186/s13049-021-00841-1 PMC7852085 33526058

[33] Zuniga A, Flores H, Lagerspetz E, Nurmi P, Tarkoma S, Hui P, Manner J. Tortoise or Hare? Quantifying the Effects of Performance on Mobile App Retention. In: Liu L, White R, editors. The World Wide Web Conference; 13.05.2019–17.05.2019; San Francisco, CA, USA. New York, New York, USA: ACM Press; 2019. pp. 2517–2528. 10.1145/3308558.3313428

[34] Sigg S, Lagerspetz E, Peltonen E, Nurmi P, Tarkoma S. Exploiting usage to predict instantaneous app popularity. ACM Trans Web. 2019;13:1–25. 10.1145/3199677.

[35] Ickin S, Wac K, Fiedler M, Janowski L, Hong J-H, Dey AK. Factors influencing quality of experience of commonly used mobile applications. IEEE Commun Mag. 2012;50:48–56. 10.1109/MCOM.2012.6178833.

[36] Baumel A, Muench F, Edan S, Kane JM. Objective user engagement with mental health apps: systematic search and Panel-Based usage analysis. J Med Internet Res. 2019;21:e14567. 10.2196/14567. 31573916 10.2196/14567 PMC6785720

[37] Lin Y-H, Chen S-Y, Lin P-H, Tai A-S, Pan Y-C, Hsieh C-E, Lin S-H. Assessing user retention of a mobile app: survival analysis. JMIR Mhealth Uhealth. 2020;8:e16309. 10.2196/16309. 33242023 10.2196/16309 PMC7728530

[38] Kirwan M, Duncan MJ, Vandelanotte C, Mummery WK. Using smartphone technology to monitor physical activity in the 10,000 steps program: a matched case-control trial. J Med Internet Res. 2012;14:e55. 10.2196/jmir.1950. 22522112 10.2196/jmir.1950 PMC3376516

[39] Vercammen S, Moens E. Cost-effectiveness of a novel smartphone application to mobilize first responders after witnessed OHCA in Belgium. Cost Eff Resour Alloc. 2020;18:52. 10.1186/s12962-020-00248-2. 33292296 10.1186/s12962-020-00248-2 PMC7673090

[40] Wade VA, Eliott JA, Hiller JE. Clinician acceptance is the key factor for sustainable telehealth services. Qual Health Res. 2014;24:682–94. 10.1177/1049732314528809. 24685708 10.1177/1049732314528809

[41] Derkenne C, Jost D, Roquet F, Dardel P, Kedzierewicz R, Mignon A, et al. Mobile smartphone technology is associated with Out-of-hospital cardiac arrest survival improvement: the first year greater Paris fire brigade experience. Acad Emerg Med. 2020;27:951–62. 10.1111/acem.13987. 32445436 10.1111/acem.13987

[42] Vilendrer S, Amano A, Brown Johnson CG, Favet M, Safaeinili N, Villasenor J, et al. An App-Based intervention to support first responders and essential workers during the COVID-19 pandemic: needs assessment and mixed methods implementation study. J Med Internet Res. 2021;23:e26573. 10.2196/26573. 33878023 10.2196/26573 PMC8139393

[43] Initiative D. e. V. D21-Digital-Index 2021/2022: Wie digital ist Deutschland? Jährliches Lagebild zur Digitalen Gesellschaft. 1st ed. Berlin; 2022.

[44] Kausmann C, Hagen C. Gesellschaftliche Bereiche des freiwilligen engagements. In: Simonson J, Kelle N, Kausmann C, Tesch-Römer C, editors. Freiwilliges engagement in Deutschland. Wiesbaden: Springer Fachmedien Wiesbaden; 2022. pp. 95–124. 10.1007/978-3-658-35317-9_6.

[45] Arriagada C, Simonson J. Freiwilliges engagement hochaltriger Menschen: beteiligung und engagementbereiche. Berlin: SSOAR, GESIS – Leibniz-Institut für Sozialwissenschaften e.V; 2021.

[46] Taramarcaz V, Herren T, Golay E, Regard S, Martin-Achard S, Mach F, et al. A short intervention and an interactive e-Learning module to motivate medical and dental students to enlist as first responders: implementation study. J Med Internet Res. 2022;24:e38508. 10.2196/38508. 35583927 10.2196/38508 PMC9161047

[47] Phung V-H, Trueman I, Togher F, Orner R, Siriwardena AN. Community first responders and responder schemes in the united Kingdom: systematic scoping review. Scand J Trauma Resusc Emerg Med. 2017;25:58. 10.1186/s13049-017-0403-z. 28629382 10.1186/s13049-017-0403-z PMC5477292

[48] Dainty KN, Vaid H, Brooks SC. North American public opinion survey on the acceptability of crowdsourcing basic life support for Out-of-Hospital cardiac arrest with the pulsepoint mobile phone app. JMIR Mhealth Uhealth. 2017;5:e63. 10.2196/mhealth.6926. 28526668 10.2196/mhealth.6926 PMC5451638

[49] Ozcan K, Jorgenson D, Richard C, Hsieh G. Designing for targeted responder models. In: Lee CP, Poltrock S, Barkhuus L, Borges M, Kellogg W, editors. CSCW ‘17: computer supported cooperative work and social computing; 25 02 2017 01 03 2017; Portland Oregon USA. New York, NY, USA: ACM; 2017. pp. 916–24. 10.1145/2998181.2998334.

[50] Brooks SC, Simmons G, Worthington H, Bobrow BJ, Morrison LJ. The pulsepoint respond mobile device application to crowdsource basic life support for patients with out-of-hospital cardiac arrest: challenges for optimal implementation. Resuscitation. 2016;98:20–6. 10.1016/j.resuscitation.2015.09.392. 26475397 10.1016/j.resuscitation.2015.09.392

[51] Cook DA, Wittich CM, Daniels WL, West CP, Harris AM, Beebe TJ. Incentive and reminder strategies to improve response rate for Internet-Based physician surveys: A randomized experiment. J Med Internet Res. 2016;18:e244. 10.2196/jmir.6318. 27637296 10.2196/jmir.6318 PMC5045523

[52] Barclay S, Todd C, Finlay I, Grande G, Wyatt P. Not another questionnaire! Maximizing the response rate, predicting non-response and assessing non-response bias in postal questionnaire studies of gps. Fam Pract. 2002;19:105–11. 10.1093/fampra/19.1.105. 11818359 10.1093/fampra/19.1.105

