# Supplementary material for: An app to keep: smartphone-based dispatch of community first responder to cardiac arrest
Source: BMC Cardiovasc Disord. 2025 Mar 7;25:159. doi: 10.1186/s12872-025-04586-y (PMC11887373; doi:10.1186/s12872-025-04586-y)
Supplement: Supplementary file 1 — Supplementary Material 1 [file 12872_2025_4586_MOESM1_ESM.pdf]

**An app to keep: smartphone-based dispatch of first responder to cardiac arrest**

Tore Marks, Bibiana Metelmann, Peter Brinkrolf, Karl Christian Thies, Klaus Hahnenkamp, Camilla Metelmann

Corresponding author: Tore Marks, Department of Anaesthesiology, University Medicine Greifswald, Greifswald, Germany, e-mail address: tore.marks@gmail.com

## Translated Questionnaire

**Survey of the „Land|Retter" after one, two and three years project time**

*„Thank you for using the Land|Retter App and for continuing to optimise the care of cardiac arrest victims in the district of Vorpommern-Greifswald.*
*[One/two/three] years after the introduction of the Land|Retter App in September 2017, we would like to ask you a few questions as part of our quality assessment process.*
*Please tick the answers that are most applicable to you in each case."*

1. When did you participate in the Land|Retter training?
    - September 2017 - August 2018
    - September 2018 - November 2019
    - December 2019 - February 2020
    - March 2020 - May 2020
    - June 2020 - October 2020
2. Do you still have the Land|Retter app installed on your phone?
    - Yes
    - No
3. How did you hear about the Land|Retter App? (Multiple answers possible)
    - Friends/family/acquaintances
    - Employers/work colleagues
    - LandRetter booth
    - Via social media/internet
    - Posters/flyers
    - Aid organisation
    - Fire brigade
    - Press
    - I can't remember
    - Other
4. Which mobile phone provider do you use?
    - Telekom / Congstar / Freenetmobile / klarmobil.de
    - Vodafone / 181 / Otelo
    - 02 / Blau / Simply / PremiumSim
    - Other
    - I don't know
5. Which operating version of the app do you use?
    - iOS (Apple)
    - Android

6. How often do you "swipe" away to close the app?
    - Several times a day
    - Daily
    - Occasionally
    - Never
7. How often do you update your Land|Retter App?
    - My phone updates automatically
    - I update the app regularly
    - I update the app occasionally
    - I never update the app
    - I don't
8. When did you install the app?
    - A few days before the training
    - A few weeks before the training
    - A few months before the training
    - I installed the app during the training day/after training
9. The app works mostly
    - Error-free
    - With minor problems
    - With major problems
10. Please describe your problems (linked free text answer; only appears if „minor“ or „major“ problems are stated)
11. How often did you have the app switched on?
    - Always
    - A few hours a day
    - A few hours a week
    - A few hours a month
    - Never
12. Do you use the app's absence/availability option?
    - Yes
    - No
13. Are there times you generally turn off the Land|Retter app/set absences?
    - Yes
    - No
14. Times where I generally turn off the Land|Retter app (linked to free text answer; text field only appears if „yes“ in question 13 is stated)
15. Do you use the app during working hours?
    - No
    - Yes
    - Sometimes
16. How often do you use the app, compared to in the past?
    - More often
    - Same frequency
    - Less often
    - Still not

17. Are there barriers to using the app? (multiple answers possible)
    - No reasons
    - Battery drainage
    - Data security
    - Mental stress caused by the permanent possibility to get dispatched
    - Concerns to get alarmed at an inconvenient time
    - Doubts regarding medical benefit
    - Legal aspects
18. Have you recommended the app to others?
    - No
    - Yes
19. During the last year my opinion regarding the *medical benefit* of the app has…
    - Improved
    - Stayed the same
    - Worsened
20. During the last year my opinion regarding the *functionality* of the app has…
    - Improved
    - Stayed the same
    - Worsened
21. How often have you been alerted so far? (excluding test alarms)
    - Never
    - Once
    - Several times
22. Have you ever declined a mission?
    - Yes
    - No
23. Why did you decline a mission? (Multiple answers possible, linked answer; only appears if „yes“ in question 22. is stated)
    - Malfunction of the app
    - The mission was not available any more
    - Deployment during working hours
    - Deployment in an inappropriate situation (e.g. going to the cinema/meeting)
    - I was looking after minors
    - I was unwell
    - I was concerned of not finding the mission site
    - I had drunk alcohol
    - I could not interrupt my activity
    - I was concerned of making a mistake during the mission
    - Other
24. Did you ever participate in a resuscitation of a cardiac arrest patient outside the project Land|Rettung?
    - Never
    - Yes, once
    - Yes, 2-5 times
    - More than 5 times

25. How confident are you performing chest compressions? (Slider bar between both answers)
    - Unsure
    - Confident
26. What do you like about the app? (Free text answer)
27. What do you dislike about the app? (Free text answer)
28. Why did you become a Community First Responder? (Free text answer)
29. Your age
    - 18-25 years
    - 26-35 years
    - 36-50 years
    - 51-65 years
    - >65 years
30. Gender
    - Female
    - Male
    - Not specified
31. What is your qualification?
    - Medical doctor
    - Pre-hospital doctor
    - Medical student
    - Nurse
    - Firefighter
    - Emergency Medical Technician
    - Paramedic 2 (three years training)
    - Paramedic 3 (two years training)
    - Paramedic 4 (three months training)
    - Paramedic 5 (one week training)
    - Company first aider
    - Medical assistant
    - First Aider
